# Infant CPAP for low-income countries: An experimental comparison of standard bubble CPAP and the Pumani system

**DOI:** 10.1371/journal.pone.0196683

**Published:** 2018-05-16

**Authors:** Markus Falk, Snorri Donaldsson, Thomas Drevhammar

**Affiliations:** 1 Department of Women's and Children's Health, Karolinska Institutet, Stockholm, Sweden; 2 Department of Anaesthesiology, Östersund Hospital, Östersund, Sweden; 3 Department of Neonatology, Karolinska University Hospital, Stockholm, Sweden; Center of Pediatrics, GERMANY

## Abstract

**Introduction:**

Access to inexpensive respiratory support to newborn infants improves survival in low-income countries. Standard bubble continuous positive airway pressure (CPAP) has been extensively used worldwide for more than 30 years. One project aimed at providing affordable CPAP is the Pumani system developed by Rice 360°. Compared to standard bubble CPAP the system has an unconventional design. The aim was to compare the Pumani system with two traditional bubble CPAP systems, focusing on in-vitro performance and safety.

**Methods:**

The Pumani system was compared to traditional bubble CPAP from Fisher & Paykel (Auckland, New Zealand) and Diamedica (Devon, United Kingdom). The systems were tested using static flow resistance and simulated breathing for a range of fresh gas flows and submersion levels.

**Results:**

There were large differences between the Pumani CPAP and the conventional bubble CPAP systems. The Pumani system was not pressure stable, had high resistance and high imposed work of breathing. It was not possible to use submersion depth to adjust CPAP without accounting for fresh gas flow.

**Discussion:**

The Pumani design is novel and not similar to any previously described CPAP system. The main mechanism for CPAP generation was resistance, not submersion depth. The system should therefore not be referred to as bubble CPAP. The clinical consequences of its pressure instability and high imposed work of breathing are not known and trials on outcome and safety are needed.

## Introduction

Being born prematurely in low-income settings is a major cause of mortality [1]. Several simple measures have been suggested as ways to improve outcome. These include antenatal corticosteroids, maintenance of body temperature, better resuscitation practice and provision of respiratory support with continuous positive airway pressure (CPAP) [2, 3]. Access to inexpensive CPAP respiratory support for preterm infants was listed by WHO in 2012 as an area in need of innovation and implementation. The report also recognises that CPAP devices that are developed need to be tested for durability, reliability and safety [4].

CPAP was introduced by Gregory in 1971 [5] and has been widely accepted as standard of care. CPAP support is recommended in The International Liaison Committee on Resuscitation (ILCOR) consensus document [6] and in the European guidelines for treatment of respiratory distress syndrome [7]. The continuous distending pressure enables the newborn infant to stabilise breathing and gas exchange, reducing the need for mechanical ventilation and the risk of lung morbidity [8, 9].

There are several methods and technical solutions to generate CPAP support to infants. Systems available today are based on expiratory resistance, expiratory bubble CPAP, variable flow generators and ventilators with CPAP mode. Resistance, pressure stability and imposed work of breathing have been suggested to be important aspects of CPAP care [10, 11]. Previous tests in mechanical lung models have shown large differences in in-vitro performance between systems [12, 13]. Systems based purely on resistance (such as T-piece resuscitators) have consistently displayed unstable CPAP with high imposed work of breathing [12, 14, 15].

Standard bubble CPAP has been extensively used worldwide for more than 30 years. The technique is well suited for provision of lower-cost robust CPAP equipment, with standardised settings and safety features, as recommended in the WHO report [4]. Several projects approaching the challenge to provide access to affordable CPAP support have been based on the simple, robust, reliable and well known bubble CPAP design.

One of these projects resulted in the Pumani CPAP system [16, 17]. It was developed for low-income countries by Rice 360° Institute for Global Health (Houston, Texas, USA) with funding from the Gates Foundation and USAID [16]. The Pumani system includes a driver unit with a built-in bubble bottle for pressure control and a single inspiratory tube connected to Hudson prongs. The Pumani design is novel and, as far as we know, has no resemblance to any previously described CPAP system.

**Fig 1:**
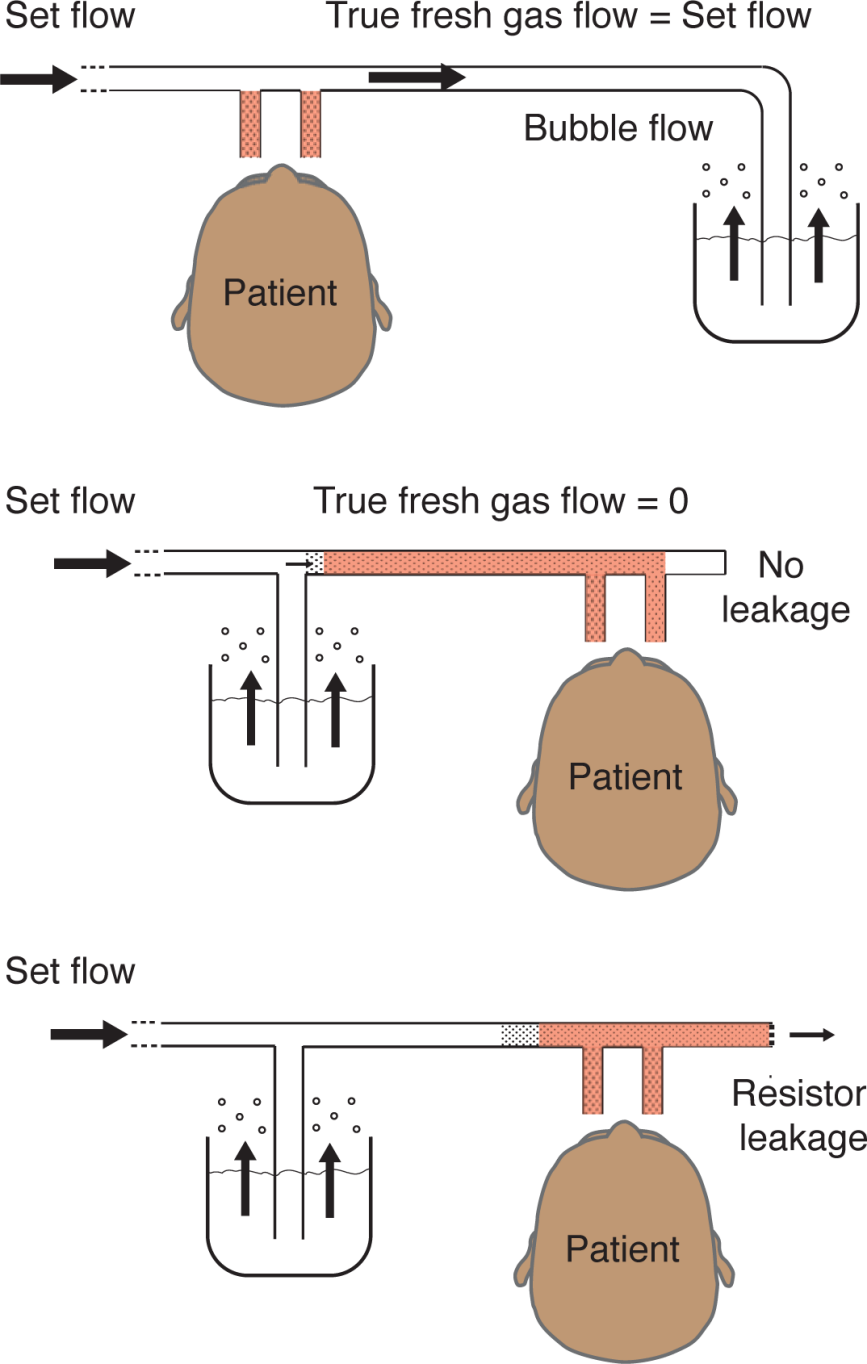
Risk of rebreathing with traditional bubble CPAP, the original Pumani and the revised Pumani CPAP. The conventional bubble CPAP (top) has the submersion bottle located on the expiratory limb. The fresh gas flow is equal to the set flow and dead-space (brown) is small (with sufficient fresh gas flow). The original Pumani (middle) had a risk for total rebreathing when there was no leakage at the patient interface. The revised Pumani (bottom) has a bleed port to ensure leakage and fresh gas flow. The illustration of the revised Pumani has been simplified by removing the narrow parts of the tubing and the bleed valve between the Y-piece and the bottle (a detailed illustration provided as Fig 2).

The most important difference to traditional bubble CPAP systems (Fig 1) is the position of the bubble bottle. It is connected to the inspiratory limb instead of the conventional position on the expiratory limb. In the Pumani system, the bottle works as a pressure release valve upstream from the patient. The flow diverted to the bubble bottle will not reach the patient and bubbling does not represent the flow that the patient receives. In a conventional bubble CPAP system bubbling always represents the true fresh gas flow.

The Pumani system has been developed over several years. The prototype system presented to the Gates foundation in 2012 had a conventional bubble CPAP design. The system was later redesigned and presented by Brown et al in 2013 with a capped expiratory limb and the bubble bottle moved to the inspiratory limb (Fig 1, Brown et al) [16]. With this design and a situation with no leakage at the interface or through the mouth, there would be total rebreathing with accumulation of carbon dioxide and subsequent respiratory failure. The researchers at Rice 360° were made aware of this risk in November 2014. In a non-randomised clinical trial by Kawaza et al this original Pumani system was compared to low flow oxygen [17]. They showed a reduction in mortality but did not have enough resources to compare with conventional bubble CPAP or measure rebreathing.

The Pumani system has since been revised with a bleed port added to the expiratory limb (previously capped). With sufficient flow through the bleed port, there is no risk of rebreathing even without leakage through the mouth or at the interface. Determining bleed port flows for the revised Pumani system is therefore needed to evaluate its safety.

The revised Pumani is a hybrid system, combining expiratory resistance (bleed port) and bubble CPAP (bottle on inspiratory limb). It has been presented as a bubble CPAP system but the novel design has not been compared to conventional bubble CPAP. Our aim was to compare the Pumani system with two traditional bubble CPAP systems, focusing on in-vitro performance and safety. Our main outcome variable was resistance represented by the pressure generated by a given flow. Our secondary outcome variable was pressure stability measured as imposed work of breathing (WOB).

## Materials and methods

The CPAP systems were evaluated for 1) effects of fresh gas flow, 2) factors affecting resistance and 3) pressure stability during simulated breathing in a mechanical lung model.

**Fig 2:**
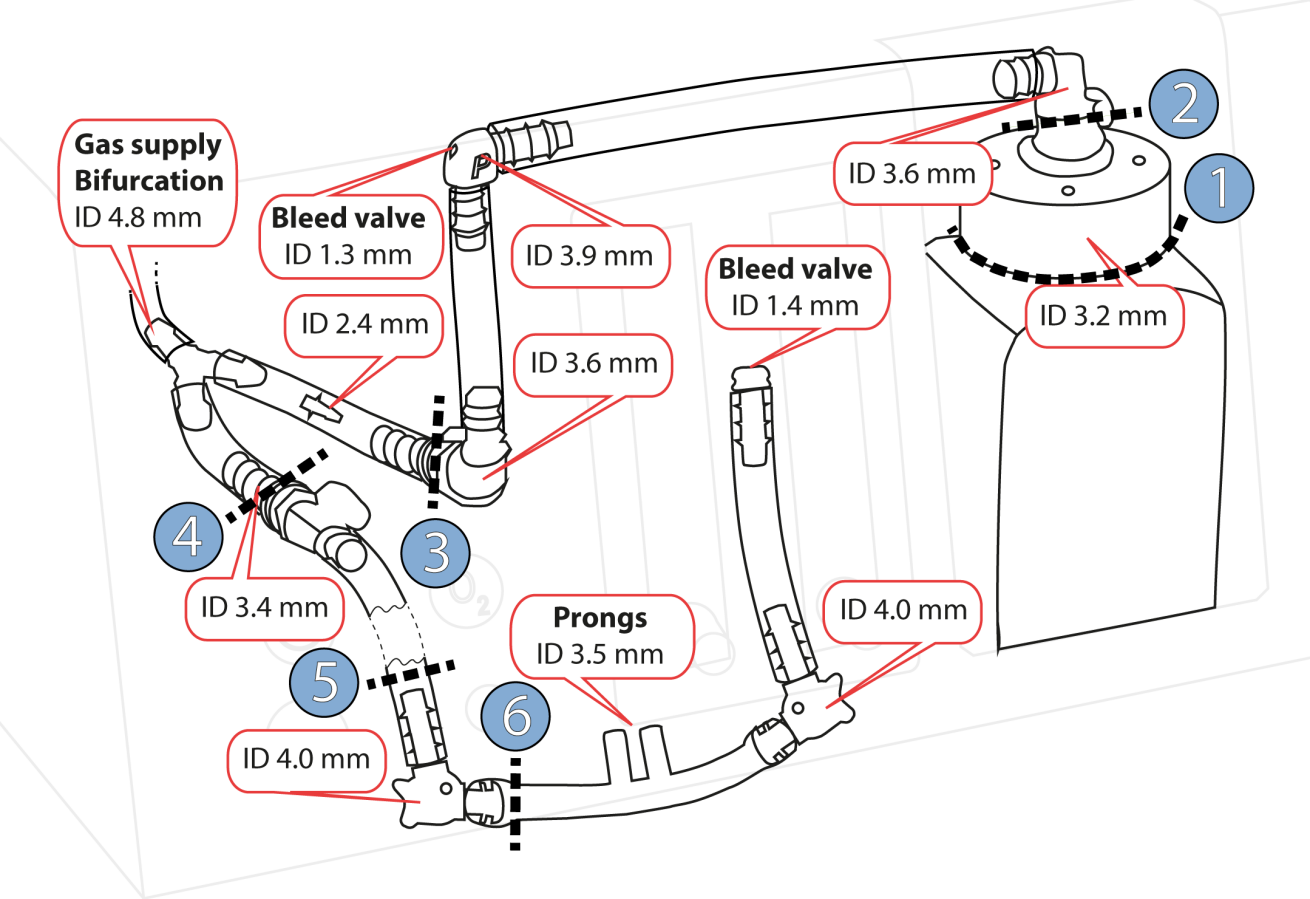
Design and dimensions of the revised Pumani. Fresh gas flow is supplied to the breathing circuit at the bifurcation. The patient is connected to the prongs and gas can leave the system through leakage at the patient, the two bleed ports and at the bubble bottle (right side). Internal diameters (ID) were estimated by the authors. Blue circle 1–6 indicate cutpoints used to determine tube resistance. A simplified illustration of the revised Pumani components is provided in Fig 1 (bottom).

The revised Pumani was compared to a Fisher and Paykel bubble CPAP (Fisher and Paykel, Auckland, New Zealand) and a Diamedica bubble CPAP (Diamedica Ltd, Devon, UK). The revised Pumani with dimensions is presented in Fig 2. The two conventional bubble CPAP systems have the bubble bottle on the expiratory limb of the patient circuit connected to the prongs. The systems were tested with prongs of approximately 4.0 mm internal diameter: Pumani size 3 (ID 3.8 mm), Fisher and Paykel 5040 (ID 4.0 mm) and Diamedica size 4 (ID 4.0 mm).

Uncut endotracheal tubes (Teleflex Incorporated, Pennsylvania, USA) size 3.0 (ID 2.9 mm, length 17,5 cm) and size 3.5 (ID 3.4 mm, length 19,8 cm) were used without CPAP as an illustration of resistance. The Neopuff T-piece resuscitation system (Fisher and Paykel, Auckland, New Zealand) was included for comparison and represents a pure resistor system with low pressure stability [18].

In all tests the resistance was represented by the generated pressure at a given flow. With increasing flows, a higher resistance generates a steeper rise in pressure. In most experiments the resistance was non-linear and it was not possible to simplify the comparison with a simple mathematical relationship. The resistance was therefore presented graphically and the complete data as supplements.

### Measurements without simulated airway flow

**Fig 3:**
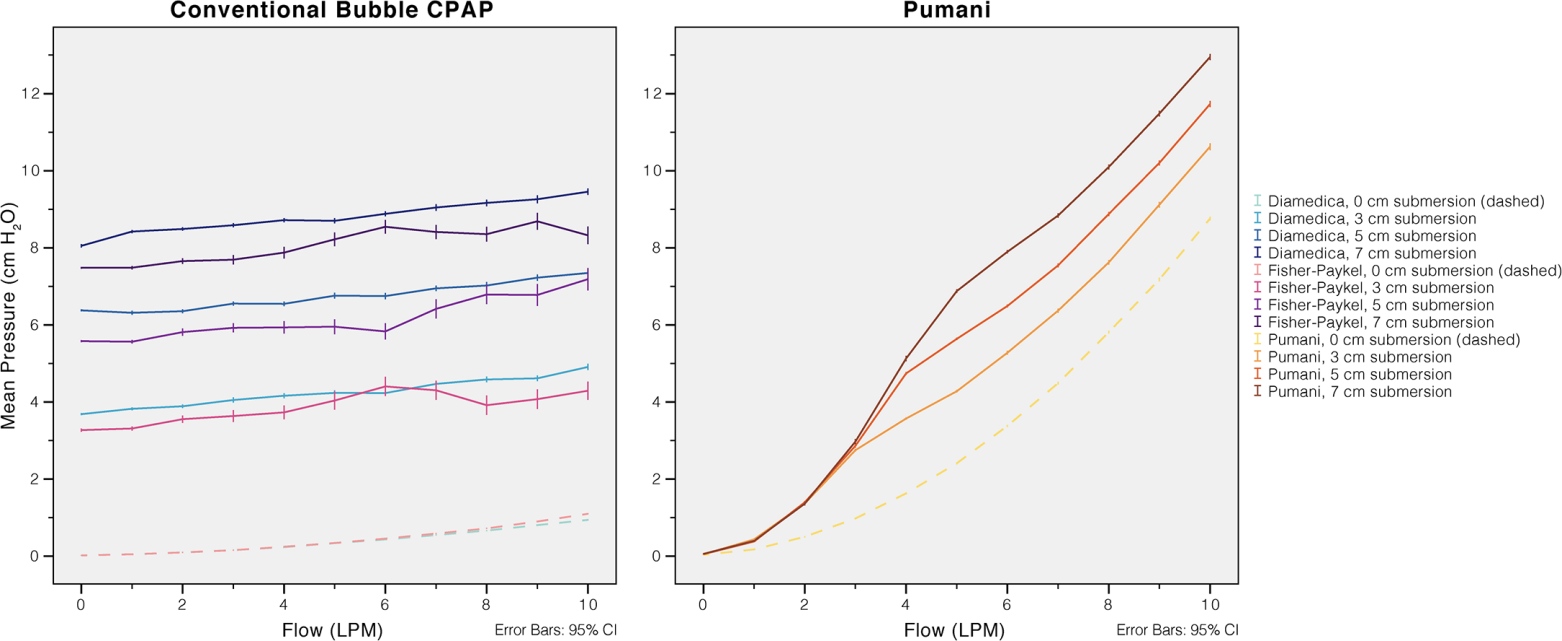
Fresh gas flow and generated CPAP. An increase in fresh gas flow resulted in higher CPAP. The conventional bubble CPAP systems were less sensitive to adjustments in fresh gas flow than the revised Pumani.

The CPAP generated by each system was measured using a slow and gradual increase in driver flow from 0 to 10 L/min with 0, 3.0, 5.0 and 7.0 cm of submersion (patient interface occluded). For the revised Pumani, bleed port flows were collected at the same time (Fig 3).

### Measurements with simulated airway flow

Resistance was measured as the change in pressure at a range of airway flows (-10 to 10 L/min). Negative flows correspond to patient inspiration and positive flows to patient expiration. The airway flow, through the patient interface, was generated by a ASL 5000 mechanical lung simulator (IngMar Medical, Pennsylvania, USA) set to complete two breathing cycles in 20 seconds. Resistance was investigated using this setup in three experiments; resistance of tubing and components, resistance with increasing submersion depths and resistance with increasing fresh gas flows.

The resistance of tubing and components was measured without any submersion or fresh gas flow. The tubing of the revised Pumani system was disconnected at six positions (Fig 2). By gradually removing more parts the resistance of each component could be measured. The Fisher and Paykel and Diamedica were only tested as complete systems. Two endotracheal tubes were added for comparison.

#### The effect on resistance and pressure stability with changing driver flows and submersion depths

Resistance for each system was measured at three levels of submersion (3.0, 5.0, 7.0 cm) with a fresh gas flow of 10 L/min and with three levels of fresh gas flow (6, 8 and 10 L/min) with a submersion depth of 5.0 cm.

#### Imposed work of breathing

Imposed work of breathing (WOB) was measured using the mechanical lung simulator connected to the prongs. The variables were collected at CPAP levels of 0 to 10 cm H_2_O (Pumani to 14 cm H_2_O) by increasing submersion depth or increasing expiratory resistance (Neopuff) over three to four minutes. The mechanical lung model was used in the non-compliant pump mode with a sinusoidal flow pattern and a flow maximum of 6 L/min, tidal volumes of 32 mL, respiratory rate of 60/min and an inspiratory-expiratory ratio of 1:1. Data was collected for each breath and analysed with the modified software supplied by the manufacturer (modified version 3.1, IngMar Medical, Pennsylvania, USA). The imposed work of breathing was calculated by integration of the pressure-volume loop for each breath (area within the loop). Measuring imposed work of breathing has been discussed by Banner [19] and the method has been used in our previous work [15, 18].

The CPAP systems were connected to the measuring equipment using 15 or 22 mm standard connectors. The prongs were connected using adhesive putty and checked for leakage. The pressure generated by the CPAP systems was measured at the prongs. Fresh gas flow was measured at the connection to the gas source. In the revised Pumani this was before the bifurcation inside the driver. All tests used dry, unheated air. Simulated airway flow was measured at the patient side of the prongs. For the revised Pumani, the bleed port flow was measured at the outlet.

Flow was measured using two SFM-3200-60-AW flow meters (SENSIRION AG, Staefa, Switzerland), each connected to a microcontroller board (Arduino Uno, Arduino, Italy).

Pressure was measured using a Honeywell sensor (40PC001B1A, Honeywell Inc. Freeport, IL, USA) connected to a DAQ (NI-6451, National Instruments, Austin, TX, USA) and was calibrated using a FLUKE VT PLUS HF (Fluke Biomedical, Everett, WA). The flow meters were calibrated using a MesaLabs Bios Defender 510 (Brandt Instruments, Prairieville, LA). Data was collected at 500 Hz using LabVIEW (LabVIEW 2015, National Instruments Corporation, Austin, TX, USA), compiled and analysed in SPSS (Ver. 24, IBM, Armonk, NY, USA). Pressures and flows were compiled in intervals of 1 cm H_2_O or 1 L/min (bins) to allow presentation of variance and linear plots.

Statistical analyses were performed using ANOVA with Bonferroni correction and mean data was presented with standard deviation (supplementary tables) or 95% confidence interval (figures). P **<** 0.05 was considered to be statistically significant.

## Results

**Fig 4:**
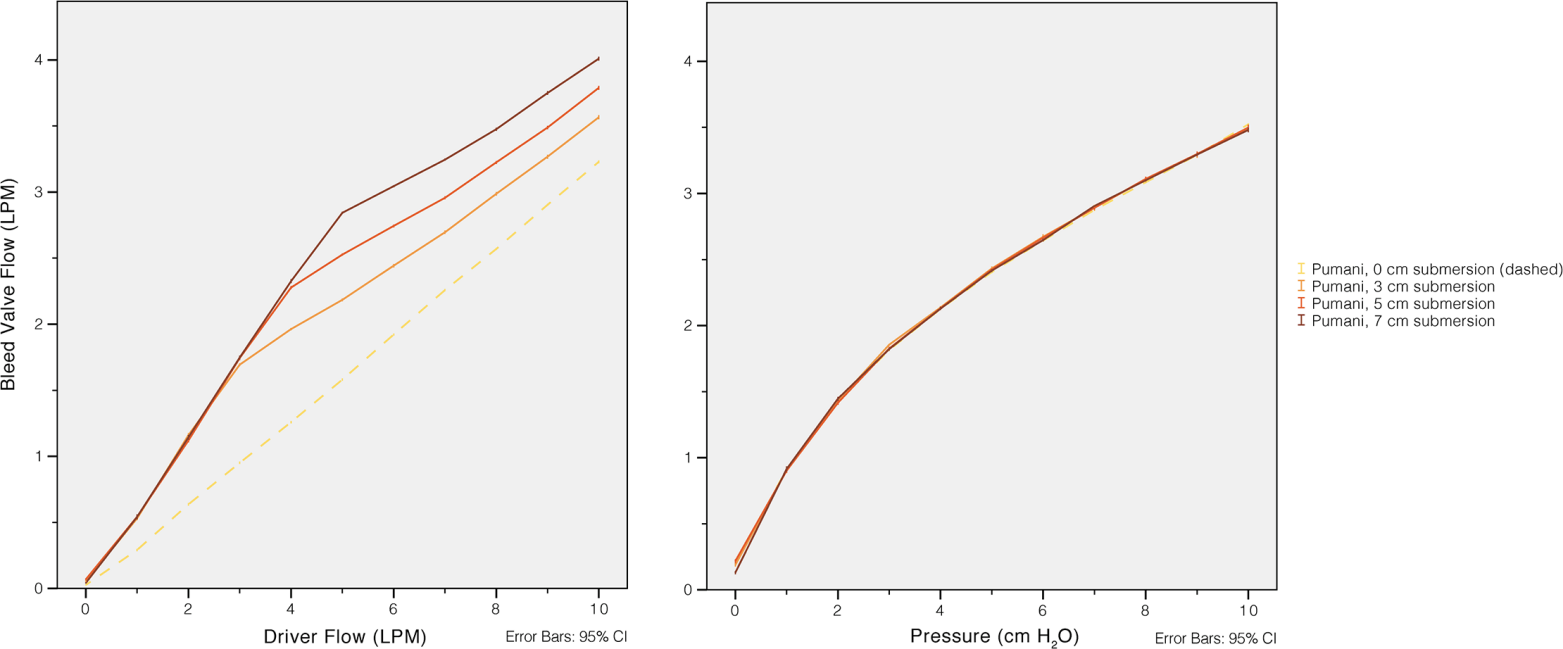
Pumani bleed port flow at different levels of fresh gas flow (left) and CPAP (right). The bleed port flow exceeds 1 L/min at fresh gas flows of approximately 2 L/min or CPAP of 1 cm H_2_O. The revised Pumani is equivalent to a ‘Mapleson A’ system and a bleed port flow higher than the minute ventilation will prevent re-breathing even if there is no leakage at the interface or through the mouth.

**Fig 5:**
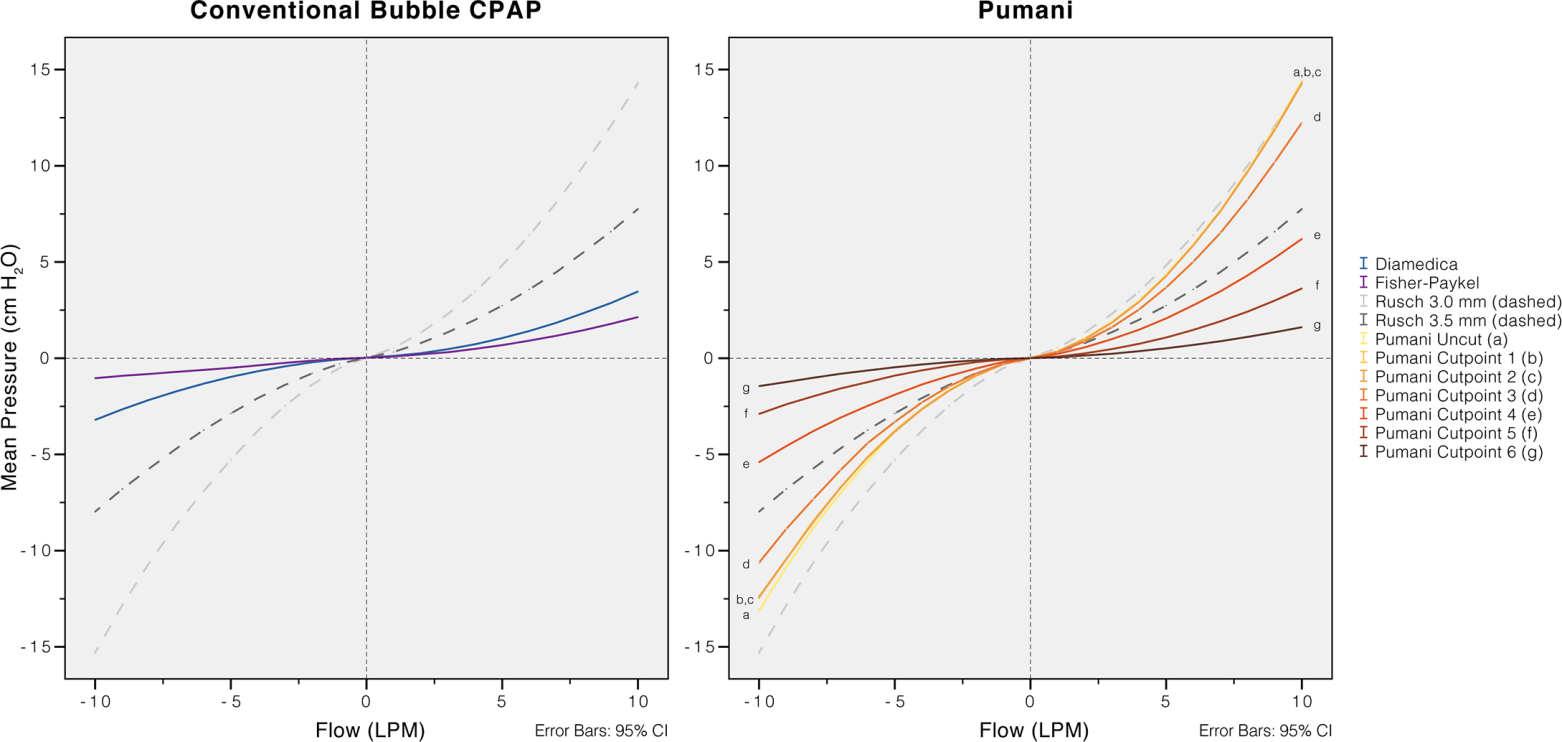
Resistance of tubing and connectors. The resistance for the revised Pumani system was comparable to an uncut endotracheal tube size 3. The resistance was reduced as parts of the Pumani tubing were disconnected. Removing the bottle and attached tubing have small effects on resistance (curves cutpoint 1 to 3 close to identical, Fig 2). A main component in the resistance was from the driver (driver removed in cutpoint 4 to 6, Fig 2) but all connectors (to the prongs) had to be removed before resistance was lower than for the two conventional bubble CPAP systems. The resistance was measured without CPAP (no submersion or fresh gas flow). Negative flows correspond to patient inspiration.

**Fig 6:**
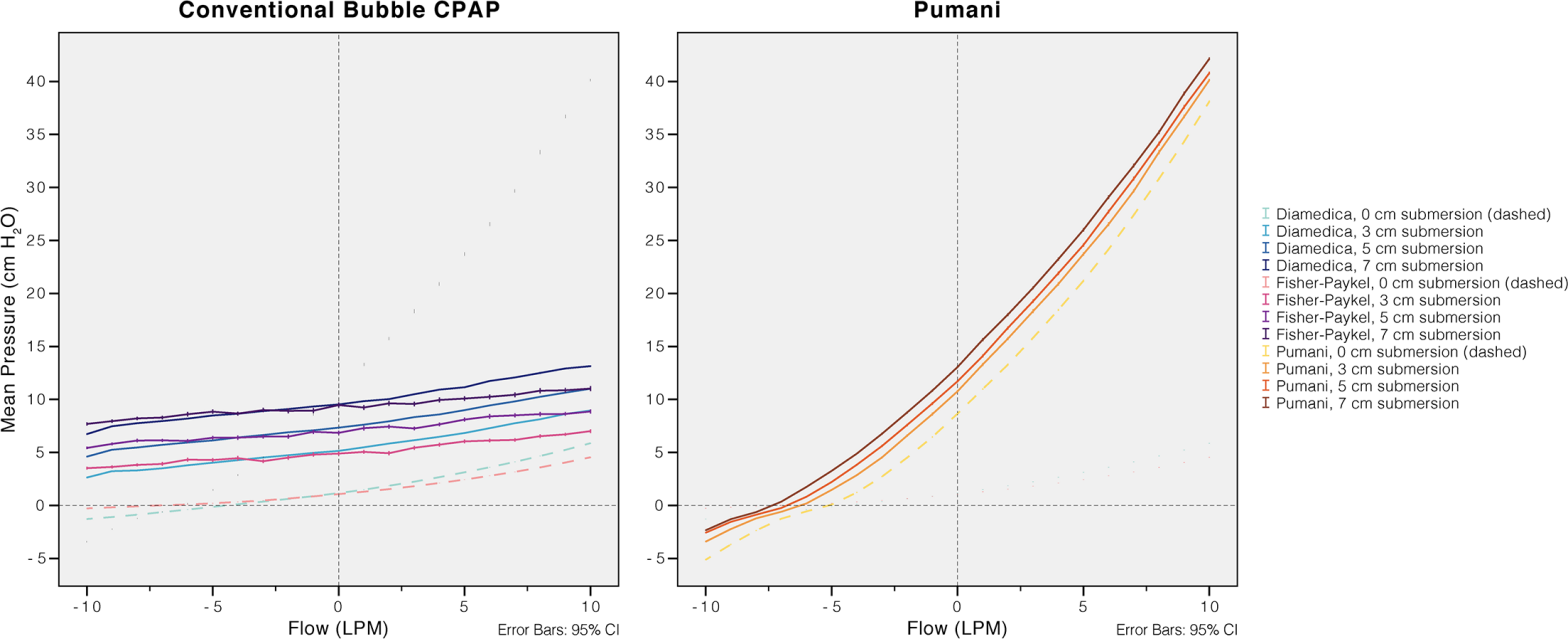
Resistance for each system with increased submersion depths and a fresh gas flow of 10 L/min. There is an increase in pressure during expiration and a decrease during inspiration. The revised Pumani has higher resistance (steeper slope) than conventional bubble CPAP systems. Simulations without submersion were included to illustrate resistance from the connectors, tubing and prongs. Negative flows correspond to patient inspiration.

**Fig 7:**
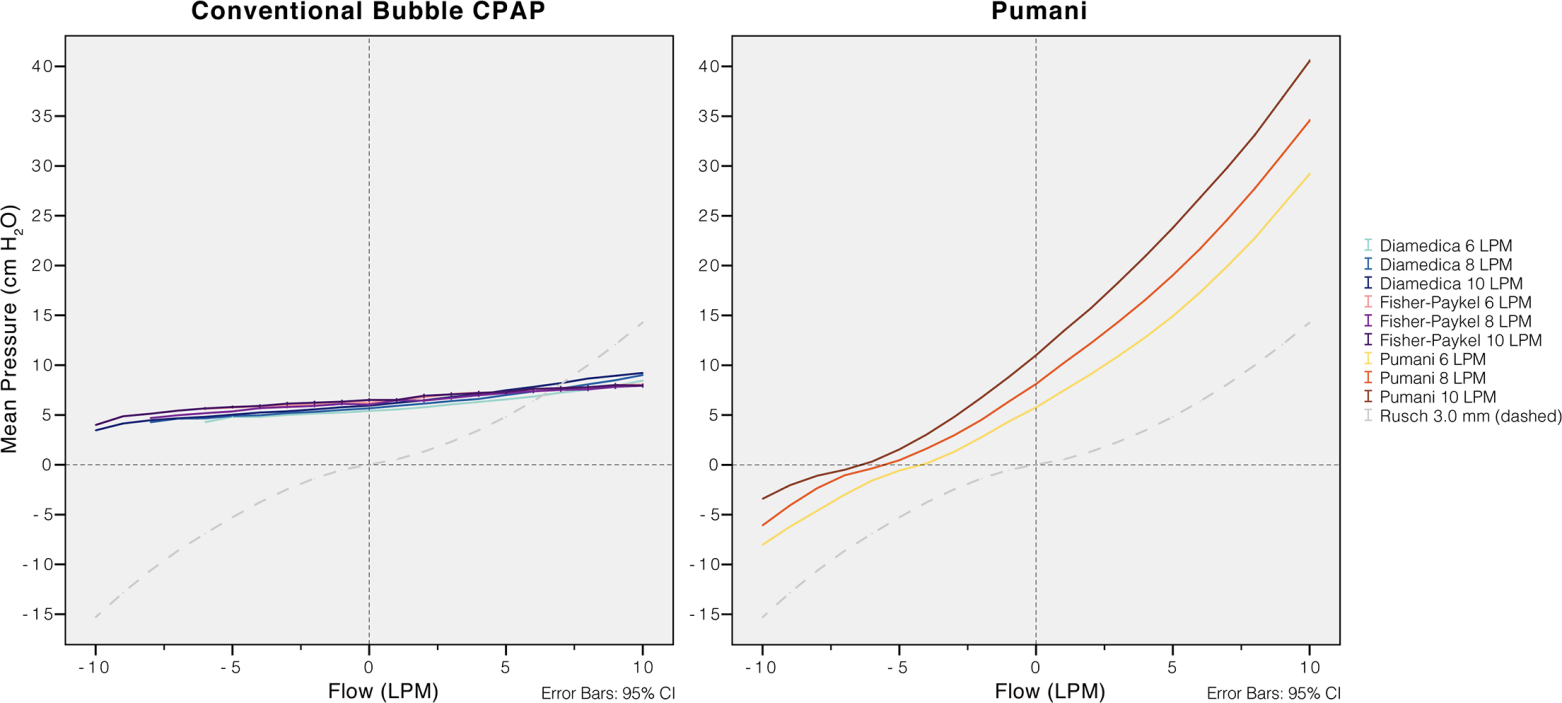
Resistance for each system with increasing fresh gas flows and a submersion depth of 5.0 cm. Due to the system resistance, with increasing fresh gas flows, CPAP (pressure at 0 L/min of simulated airway flow) increased slightly in the Diamedica and the Fisher & Paykel and more pronounced in the revised Pumani. For all systems the resistance (slope) was similar at different levels of fresh gas flows. An uncut endotracheal tube size 3 (no fresh gas flow) was included for comparison. Negative flows correspond to patient inspiration.

**Fig 8:**
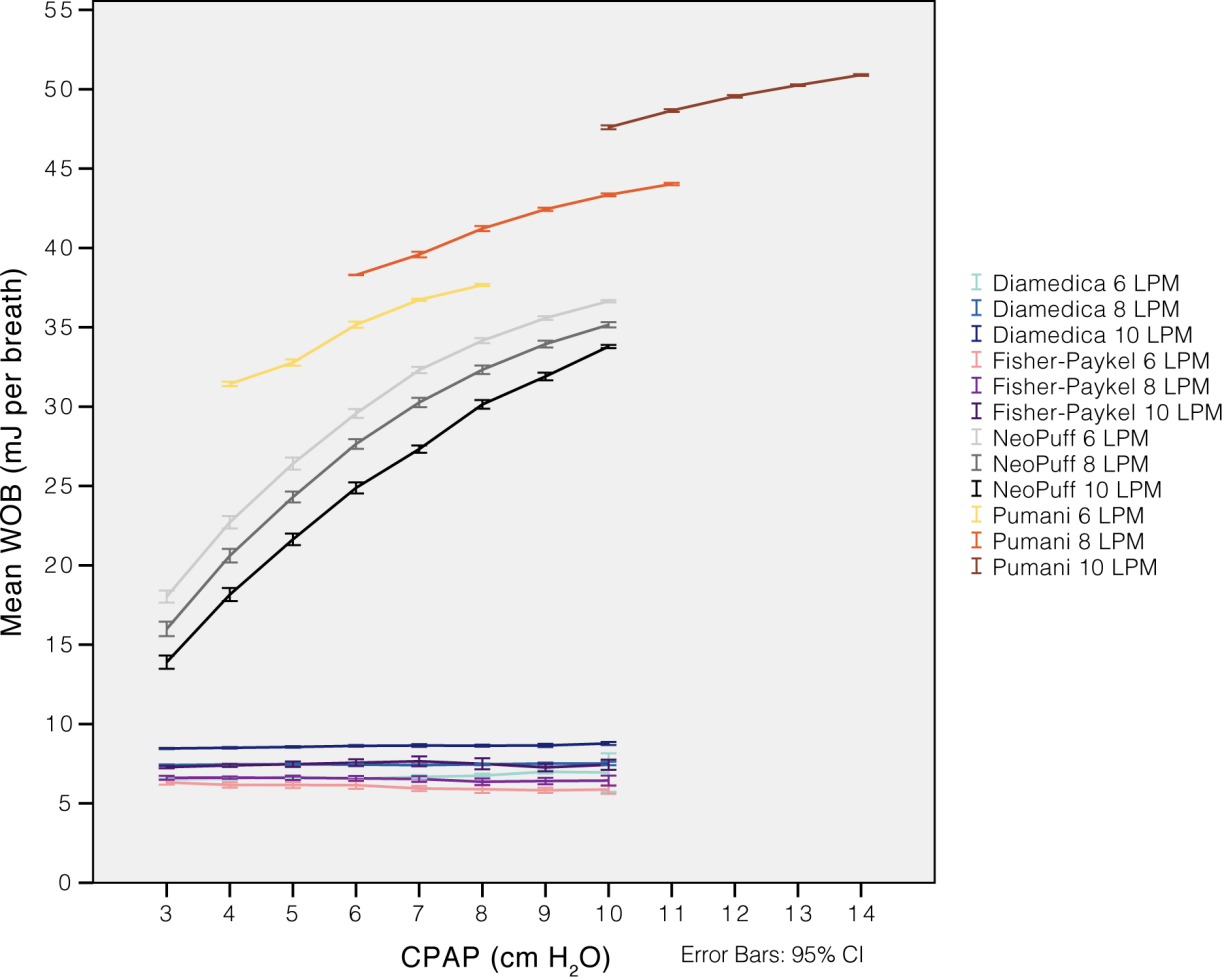
Imposed work of breathing at increasing CPAP levels. The Pumani system was tested with a constant fresh gas flow (6, 8 or 10 L/min) and increasing submersion depths from 0 to 8.5 cm. This increase resulted in CPAP rising with approximately 4 cm H_2_O. The imposed work of breathing was higher than for the other CPAP systems. The Neopuff T-piece resuscitation system was included for comparison (tested without interface). Simulations were performed with a 32 mL symmetrical and sinusoidal flow pattern (I:E 1:1, flow maximum 6 LPM, respiratory rate 60).

All systems were tested as intended and large differences between systems were found. The results are presented in Figs 3–8 with numerical data and statistical comparisons in S1–S6 Tables. In summary, for the revised Pumani submersion depth could not be used to adjust or control CPAP level without accounting for fresh gas flow. Even at low levels of CPAP the bleed valve flow was adequate to avoid re-breathing. The resistance and imposed work of breathing was high.

### Effect of submersion

For the conventional bubble CPAP systems, the delivered CPAP was mainly dependent on submersion depth with small effects of variations in fresh gas flow. The Pumani did not show this relation. Instead, it showed a steep increase in delivered pressure with increased fresh gas flow. This increase was present in both simulations with and without submersion (Fig 3).

### Bleed valve flow

The revised Pumani bleed port flow was directly related to CPAP pressure and was therefore dependent on fresh gas flow and submersion (Fig 4). Even at low CPAP levels the flow exceeded 1 L/min; As an example, the leakage flow was 1,69 (±0,14) L/min at 3 L/min of fresh gas flow and a submersion depth of 3.0 cm.

### Resistance and imposed work of breathing

The resistance of the revised Pumani system tubing, without fresh gas flow or submersion, was higher than in both conventional bubble CPAP systems (Fig 5). The resistance was higher than an uncut size 3.5 endotracheal tube. Disconnecting parts of the revised Pumani tubing reduced resistance. The Pumani prongs without tubing had similar resistance as the complete Fisher and Paykel system. The Fisher and Paykel had lower resistance than the Diamedica bubble CPAP system. The resistance of the revised Pumani was not affected by altering submersion depth (Fig 6) or fresh gas flow (Fig 7) and visually, the plots had similar shape. The revised Pumani had higher imposed work of breathing than the conventional bubble CPAP systems and the Neopuff resuscitator (true resistor system). For the Pumani, the effect on CPAP obtained by increasing submersion depth from 0 to maximum was limited.

## Discussion

Providing access to CPAP for newborn infants in low-income settings is both important and challenging. Bubble CPAP has major cost advantages compared to CPAP provided by more expensive techniques such as variable flow systems or ventilators. The combination of low price, robust technique, extensive clinical experience and no need for pressure monitoring makes bubble CPAP an ideal candidate for use in low-income settings. The pressure stability of conventional bubble CPAP is also equal or better compared to many ventilators and some variable flow CPAP generator systems [12, 13]. New systems developed for use in low-income settings should therefore be compared to conventional bubble CPAP.

We have compared the revised Pumani system with two conventional bubble CPAP systems. All systems are in clinical use with the Fisher and Paykel system frequently used in high-income countries. The Pumani CPAP is different to a conventional bubble CPAP system with the bottle being positioned on the inspiratory tubing (Fig 1).

In the original Pumani design the expiratory limb was capped which introduced a risk of rebreathing to the patient. The system would only be safe to use if there was leakage at the prongs or through the mouth. In a patient with no leakage there would be total rebreathing and accumulation of carbon dioxide. This original Pumani system was revised and the capped expiratory limb was replaced with a bleed port. The revised system has at least three differences to the system described by Brown in 2013; 1) addition of a bleed port on the expiratory limb 2) an additional bleed port on the tubing to the bottle and 3) a restrictor added on the same tube (inside the driver) (Fig 2). The performance of the original system was not tested in this manuscript and there is limited information on its resistance and pressure stability. Leakage is likely to have protected against rebreathing in patients treated with the original Pumani system. A trial by Huckstadt et al measuring relative leakage showed large leakage in the majority of patients [20]. The non-randomised trial performed by Kawaza et al used the original Pumani system [17]. These results cannot be translated to the revised Pumani system without extreme caution.

The level of CPAP delivered by the revised Pumani system is sensitive to changes in fresh gas flow. Adjusting the flow is more important than using a correct submersion depth. This may confuse the users since traditional bubble CPAP level can be predicted by submersion depth and is less dependent on fresh gas flow (Fig 2). In conventional bubble CPAP systems fresh gas flow is adjusted to meet peak inspiratory flows and to compensate for leakage. The Pumani system does not operate like conventional bubble CPAP systems and submersion depth alone cannot be used to predict or adjust the CPAP level. This could have major clinical implications.

The bleed port was introduced to mitigate the risk of rebreathing. The risk of rebreathing for semi-open systems was analysed by Mapleson [21] and the revised Pumani system would be equivalent to a ‘Mapleson A’ system during spontaneous breathing. There is no rebreathing in a ‘Mapleson A’ system with fresh gas flows higher than the minute ventilation. The minute ventilation in small infants will be lower than the bleed port flow even at low levels of CPAP (Fig 4). As intended, the bleed port therefore eliminates the risk of rebreathing.

The pressure stability of the revised Pumani system was investigated using static and dynamic simulations. They showed that the resistance was higher than for conventional bubble CPAP systems and also higher than breathing through an un-cut endotracheal tube size 3.5. The resistance was related to the tubing and connectors (Fig 5) and was present even with no submersion. The high resistance results in the delivery of pressure unstable CPAP and high imposed work of breathing (Fig 8).

### Limitations

Mechanical simulations are artificial and should not replace clinical trials. However, data on performance could not have been obtained in patients. The original Pumani system could not be tested since it has been discontinued. The risk of rebreathing was assessed theoretically by widely recognized principles originally described by Mapleson [21].

All systems were tested with piped, centrally supplied air. The CPAP drivers were not evaluated since this was beyond the aim of this study. Disconnecting the drivers and using piped air has no effect on resistance, pressure stability or imposed work of breathing. Driver variation in fresh gas flow or inaccuracy in flow level reading would affect the delivered CPAP in a clinical setting.

The high resistance of the revised Pumani system generated very high pressures on expiration. These pressures are not likely to occur in infants since leakage would protect the infant. The infant is also likely to change its breathing pattern resulting in reduced peak expiratory flows.

The breathing pattern used was a sinusoidal symmetrical flow pattern with a maximum flow of 6 L/min and a rate of 60 breaths per minute. This artificial pattern was preferred over recordings from real infants to allow for easy reproduction. The systems were only tested with prongs of one size. One would expect that smaller prongs would further increase resistance, reduce pressure stability and increase imposed work of breathing for all systems.

The Diamedica system was tested using standard wide bore tubing and bubble CPAP prongs (similar to Hudson prongs). Using alternative circuits with narrow bore tubing or connecting the prongs using a Y-piece have been suggested by the manufacturer and discussed with the authors. These unconventional designs were not tested but modifications of bubble CPAP with high resistance tubing, prongs or use of Y-piece connectors have risks of major loss of performance or could increase dead space.

There are other examples of modifications to conventional bubble CPAP aimed for use in developing countries [22–24]. These systems were designed to mainly be cost effective but do not specify any dimensions or assess the risk of rebreathing.

### Clinical implications and future

The importance of delivering CPAP with low resistance (low imposed work of breathing) remains to be investigated in large clinical trials. Also, clinical consequences of inability to adjust or accurately control CPAP level is not known.

The extensive experience with conventional bubble CPAP should be regarded as a major advantage. Therefore, when CPAP systems are developed for use in low-income settings, performance and safety should be equal or higher than in conventional bubble CPAP systems. Any modification needs to be carefully evaluated to preserve safety, efficacy and to assure that previous evidence and experience from using bubble CPAP is applicable. Bore dimensions and Y-piece connectors are important aspects of CPAP performance and need to be tested when making alterations on classical designs.

Even if clinical consequences of exposing patients to the risk of rebreathing or to high imposed work of breathing is not known, it seems logical to avoid both risks if possible. The current Pumani design with a bleed port eliminates the risk of rebreathing but exposes patients to high resistance and difficulties in adjusting CPAP by submersion depth. Large clinical trials would be needed to compare the Pumani system to conventional bubble CPAP systems in relation to effects and outcome. A source of bias and possible safety concern for the revised Pumani system is that submersion depth does not predict CPAP level. Tight control of fresh gas flow would be essential to generate comparable CPAP levels in treatment arms.

### Conclusions

In our in-vitro tests the Pumani system had high resistance, low pressure stability and high imposed work of breathing compared to the conventional bubble CPAP systems. The Pumani system generated CPAP mainly by resistance. The CPAP level was dependent on fresh gas flow and could not be controlled by submersion depth alone. The Pumani system should therefore not be referred to as bubble CPAP. The clinical consequences of our findings are not known. The large differences in performance compared to conventional bubble CPAP may affect outcome.

## Supporting information

S1 Table Generated pressure with fresh gas flows 0 to 10 L/min at fixed submersion depths (0, 3.0, 5.0, 7.0 cm). Pressure measured at the patient interface and presented as means with standard deviation (SD) for flows using 1 L/min intervals (bins). Statistical comparison was performed for each flow category, including all systems and submersion depths. Letters (a-z, A-G) indicate no statistical difference (ANOVA with Bonferroni correction). For flow category 0 L/min, multiple comparisons were not significant (indicated by *).
(XLSX)

S2 Table Pumani system bleed valve flow with fresh gas flows 0 to 10 L/min at fixed submersion depths (0, 3.0, 5.0, 7.0 cm). Flow measured at bleed valve exit port and presented as means with SD for flows and CPAP using 1 L/min and 1 cm H_2_O intervals (bins). Statistical comparison was performed for each flow and pressure category, including all submersion depths. Letters (a-z, A-Z, α-δ) indicate no statistical difference (ANOVA with Bonferroni correction). For flow category 0 L/min and pressure category 0 cm H_2_O, multiple comparisons were not significant (indicated by *).
(XLSX)

S3 Table Pressure generated for flows -10 to 10 L/min through complete and disassembled systems. Pressure measured at the patient interface and presented as means with standard deviation (SD) for flows using 1 L/min intervals (bins). Statistical comparison was performed for each flow category, including all systems and submersion depths. Letters (a-z, A-Z, α-ξ) indicate no statistical difference (ANOVA with Bonferroni correction). For flow categories -1, 0 and 1 L/min, multiple comparisons were not significant (indicated by *).
(XLSX)

S4 Table Pressure generated for added flows -10 to 10 L/min with a fresh gas flow of 10 L/min at fixed submersion depths (0, 3.0, 5.0, 7.0 cm). Pressure measured at the patient interface and presented as means with standard deviation (SD) for flows using 1 L/min intervals (bins). Statistical comparison was performed for each flow category, including all systems and submersion depths. Letters (a-z) indicate no statistical difference (ANOVA with Bonferroni correction). For flow categories -1, 0 and 1 L/min, multiple comparisons were not significant (indicated by *).
(XLSX)

S5 Table Pressure generated for flows -10 to 10 L/min with fixed driver flows (0, 6, 8, 10 LPM). Pressure measured at the patient interface and presented as means with standard deviation (SD) for flows using 1 L/min intervals (bins). In the Pumani and Fisher-Paykel data had to be truncated to avoid negative pressures and water leaving the bottle. Statistical comparisons were performed for each flow category, including all systems and submersion depths. There were small differences between the two traditional bubble CPAP systems resulting in a large number of non significant comparisons (75 in total). These were not indicated in the table. In comparisons involving the Pumani, letters (a-f) indicate no statistical difference (ANOVA with Bonferroni correction).
(XLSX)

S6 Table Work of breathing for each system at different levels of CPAP. Total imposed work of breathing (WOB) measured for individual breaths and presented as means with standard deviation (SD) compiled in 1 cm H_2_O intervals (bins). Statistical comparisons were performed for each flow bin, including all systems and submersion depths. Letters (a-z, A-D) indicate no statistical difference (ANOVA with Bonferroni correction).
(XLSX)
