# Correction: Infant CPAP for low-income countries: An experimental comparison of standard bubble CPAP and the Pumani system

**DOI:** 10.1371/journal.pone.0201083

**Published:** 2018-07-17

**Authors:** Markus Falk, Snorri Donaldsson, Thomas Drevhammar

Fig 2 is incorrect. The authors have provided a corrected version here.

**Fig 2:**
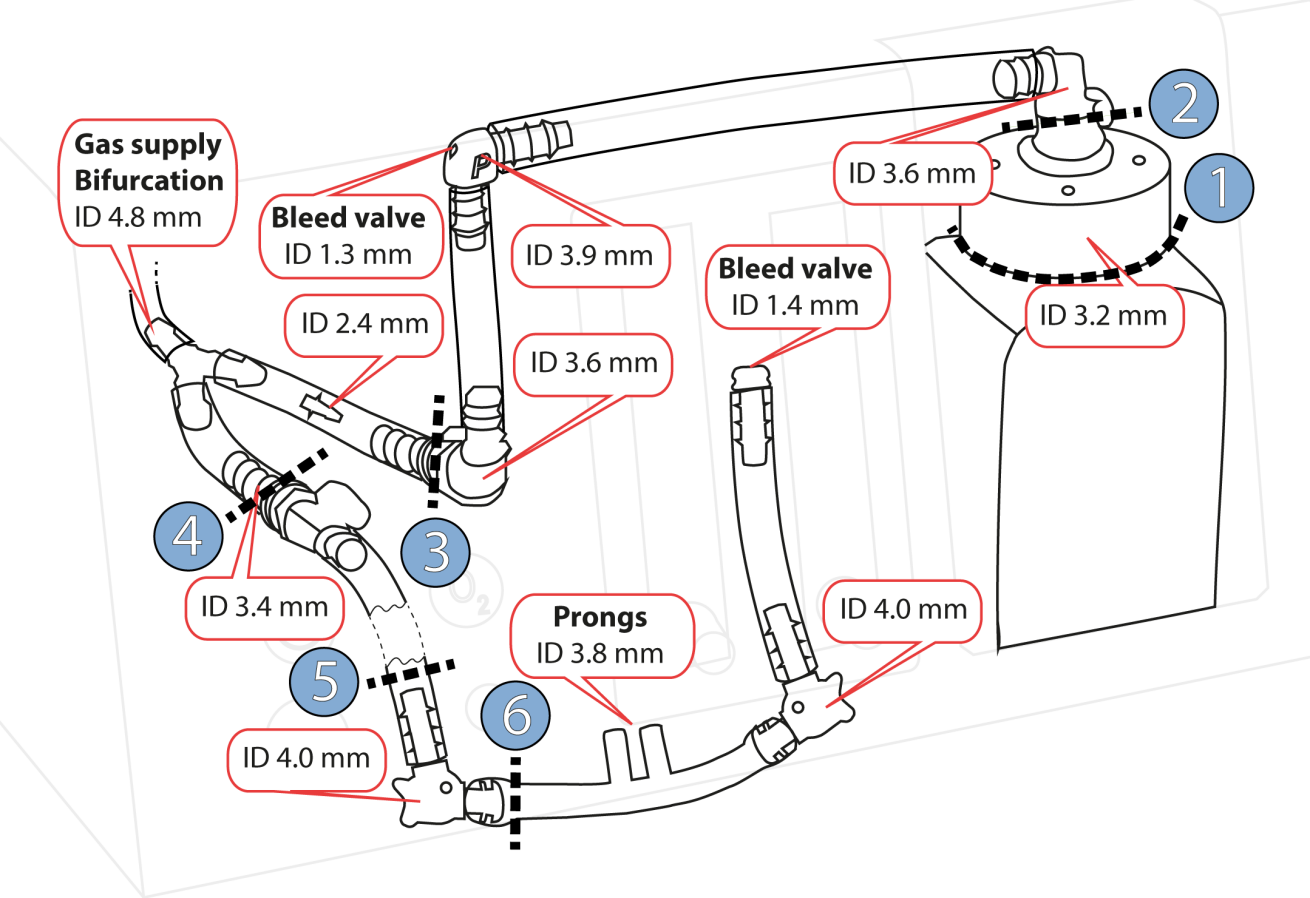
Design and dimensions of the revised Pumani. Fresh gas flow is supplied to the breathing circuit at the bifurcation. The patient is connected to the prongs and gas can leave the system through leakage at the patient, the two bleed ports and at the bubble bottle (right side). Internal diameters (ID) were estimated by the authors. Blue circle 1–6 indicate cutpoints used to determine tube resistance. A simplified illustration of the revised Pumani components is provided in Fig 1 (bottom).
